# Prognostic relevance of left atrial function and stiffness in heart failure with preserved ejection fraction patients with and without diabetes mellitus

**DOI:** 10.3389/fcvm.2022.947639

**Published:** 2022-09-14

**Authors:** Shuangshuang Zhu, Yixia Lin, Yanting Zhang, Guohua Wang, Mingzhu Qian, Lang Gao, Mengmeng Ji, Mingxing Xie, Yuman Li, Li Zhang

**Affiliations:** ^1^Department of Ultrasound Medicine, Union Hospital, Tongji Medical College, Huazhong University of Science and Technology, Wuhan, China; ^2^Clinical Research Center for Medical Imaging in Hubei Province, Wuhan, China; ^3^Hubei Province Key Laboratory of Molecular Imaging, Wuhan, China; ^4^Department of Cardiovascular Surgery, Union Hospital, Tongji Medical College, Huazhong University of Science and Technology, Wuhan, China

**Keywords:** heart failure with preserved ejection fraction, type 2 diabetes mellitus, left atrial function, stiffness, prognosis

## Abstract

**Background:**

Although the left atrium (LA) plays a key role in the pathophysiology and disease progression of heart failure with preserved ejection fraction (HFpEF), the impact of type 2 diabetes mellitus (T2DM) on LA function and stiffness in HFpEF patients remains unclear. Furthermore, the prognostic value of different phases of LA function and stiffness is less well-established in HFpEF patients.

**Methods:**

This study prospectively enrolled 164 HFpEF patients who were in sinus rhythm at the time of echocardiography, including 61 (37%) HFpEF patients with T2DM. LA reservoir, conduit, and pump function were assessed using two-dimensional volume indices and speckle tracking echocardiography. The LA stiffness was calculated as the ratio of early mitral inflow velocity-to-early annular tissue velocity (E/e’) and LA reservoir function. The primary end point was a combined outcome of heart failure hospitalization or death.

**Results:**

Left atrium reservoir function [measured by peak LA strain (LAS-peak)] and LA pump function (measured by LAS-active) remained significantly lower in the HFpEF patients with T2DM compared with those without T2DM, even after adjustment for potential confounders. In addition, the LA stiffness of HFpEF patients with T2DM was higher than those without T2DM. After a median follow-up of 13.7 months, 46 patients (28.1%) reached the composite end point. LAS-peak (hazard ratios: 0.88; 95% confidence interval: 0.81–0.95; *P* = 0.001) was significantly associated with the risk of heart failure hospitalization or death after adjusting for demographic and clinical characteristics, LV global longitudinal strain, E/e’, and LA volume index. In contrast, other LA function and stiffness parameters did not independently predict the risk of adverse events. Kaplan-Meier analysis showed that HFpEF patients with T2DM and low LAS-peak (<27.2%) had a significantly increased risk of heart failure-related hospitalization or death (log-rank *P* < 0.001).

**Conclusion:**

Left atrium reservoir and pump function are impaired, whereas LA stiffness is increased in HFpEF patients with T2DM compared with those without T2DM. LAS-peak is a powerful predictor of adverse clinical outcomes and may be crucial for risk stratification in HFpEF patients with and without T2DM.

## Introduction

Heart failure with preserved ejection fraction (HFpEF) is increasingly becoming a global health problem (1, 2). Type 2 diabetes mellitus (T2DM) is a frequent comorbidity of HFpEF (approximately 33–40%) (3–5). Moreover, T2DM significantly increases the risk of hospitalizations and mortality in HFpEF patients (6–8). It has been suggested that T2DM plays a central pathophysiological role in the development of HFpEF. Although the underlying mechanisms of this relationship are unclear, HFpEF patients with diabetic are more likely to have left ventricular (LV) hypertrophy, LV systolic and diastolic dysfunction, and left atrial (LA) enlargement (9, 10). The influence of T2DM on LA remodeling has been previously recognized in patients without symptomatic cardiovascular disease (11). However, the impact of T2DM on LA function and stiffness in HFpEF patients remains unclear (12).

The LA plays an integral role in the pathophysiology and disease progression of HFpEF (13–21). The LA size is independently associated with an increased risk of morbidity and mortality in HFpEF (13, 14). The different phases of LA function can be obtained using two-dimensional (2D) speckle tracking echocardiography (STE) which is a helpful tool for direct measurement of intrinsic LA myocardial deformation. 2D-STE is less dependent on loading conditions and geometric assumptions and has high feasibility and reproducibility (22, 23). Previous studies demonstrated the impairment of LA function in HFpEF compared with normal individuals (15–21, 24). However, LA function in HFpEF patients with and without T2DM has not been well-investigated. Furthermore, there is limited data on the prognostic relevance of LA function in HFpEF patients (25, 26).

The LA stiffness has been recognized as a novel method that estimates LA compliance (27). In addition, studies reported that LA stiffness is a strong independent predictor of recurrence after ablation (28) and is associated with poor clinical outcomes in patients with heart failure and reduced ejection fraction (29, 30). However, LA stiffness in HFpEF with and without T2DM has not been described, and its predictive value has also been unknown.

Accordingly, we aimed to (1) compare LA function and stiffness between HFpEF patients with and without T2DM and further investigate the impact of T2DM on LA function and stiffness in HFpEF patients and (2) determine the independent prognostic significance of LA function and stiffness in HFpEF patients, after adjustment for demographic and clinical characteristics, LV parameters, and LA size.

## Methods

### Study population

We prospectively enrolled 164 consecutive HFpEF patients between January 2021 and May 2021 at Union Hospital in Wuhan, China. HFpEF was defined according to the guidelines of the European Heart Journal (2016): (1) typical symptoms and/or signs of HF; (2) elevated B-type natriuretic peptide (BNP) > 35 pg/mL and/or N-terminal pro-B-type natriuretic peptide (NT-proBNP) > 125 pg/mL; and (3) echocardiographic LV ejection fraction (LVEF) ≥50% accompanied by either (a) diastolic dysfunction (ratio of peak early diastolic filling velocity (E) to early diastolic mitral annular velocity (e’) > 15) or (b) LA enlargement (left atrial volume index (LAVI) > 34 ml/m^2^) (31). Patients were excluded if they had atrial fibrillation, severe valvular disease, chronic obstructive pulmonary disease, congenital heart disease, acute coronary syndrome, pericardial disease, or poor image quality. This study was approved by the Ethics Committee of Wuhan Union Hospital, Tongji Medical College, Huazhong University of Science and Technology and was performed in accordance with the Declaration of Helsinki (Ethics No. 0650-01). Furthermore, we obtained written informed consent from all participants.

### Clinical characteristics

We collected the following data from all study participants: demographics, New York Heart Association (NYHA) functional class, comorbidities, medications, vital signs, body mass index (BMI), and laboratory data, including creatinine, BNP, NT-proBNP, total cholesterol, triglyceride, high-density lipoprotein, and low-density lipoprotein. T2DM was defined as the presence of the clinical diagnosis (fasting plasma glucose ≥ 7 mmol/L or glycated hemoglobin ≥ 6.5%) or a self-reported history of diabetes mellitus and/or receiving antidiabetic therapy (32).

### Echocardiographic examination

A comprehensive echocardiographic examination was acquired using the commercially available system (EPIC 7C, Philips Medical Systems, Andover, United States) with S5-1 and X5-1 transducers. All echocardiographic images were recorded in a native DICOM format. All echocardiographic measurements were performed according to the recommendation of the American Society of Echocardiography (33). Conventional echocardiographic measurements mainly included LV end-diastolic and end-systolic volume, mitral inflow propagation, mitral annular relaxation velocities, LA volume, and LVEF. LVEF was calculated using the biplane Simpson’s method in the apical 2-and 4-chamber views.

### Left atrium functions and stiffness analysis

Based on previous validated studies and guidelines of the American Society of echocardiography/EACVI, LA, and LV deformation was performed using commercially available VIS (2D Cardiac Performance Analysis, TomTec Imaging Systems, Unterschleissheim, Germany) (34). Analyses of LA strain were performed in the apical four- and two-chamber views, and LV strain was obtained from the apical four-, three-, and two-chamber views. The most suitable cardiac cycle was chosen for each view. The reference point was set at the beginning of the QRS complex. The LA and LV endocardial borders were traced at the end-diastolic frame. The accuracy of tracking was visually confirmed throughout the cardiac cycle and confirmed from the morphology of the strain curves. If necessary, the region of interest was readjusted. Speckles were tracked by the software frame using the frame during the course of 1 cardiac cycle. LV global longitudinal strain was calculated as the average LV longitudinal strain across the 16 segments obtained using the apical four-, three-, and two-chamber views.

From LA speckle tracking analysis, the LA function was estimated using volumes and strain indices calculated as the average across the apical four- and two-chamber views. LA reservoir function was assessed using the peak LA strain (LAS-peak) and the total LA emptying fraction (LAEF-total). LA pump function was evaluated using LAS-active: LA strain at the onset time of the P wave, and LAEF-active: (LA volume at the onset time of the P wave–LA minimum volume)/LA volume at the onset time of the P wave. Left atrial conduit function was estimated using LAS-passive: LAS-peak–LAS-active, and LAEF-passive: (LA maximum volume- LA volume at the onset time of the P wave)/LA maximum volume. The LA stiffness was calculated as the ratio of early diastolic mitral inflow velocity-to-early diastolic mitral annular tissue velocity (E/e’) and LA reservoir function (LAS-peak and LAEF-total) (35).

### Interobserver and intraobserver reproducibility

Intraobserver and interobserver variability of LA strain was estimated in 20 randomly selected subjects and evaluated by intra-class correlation coefficient (ICC) and Bland-Altman analysis. Intraobserver variability was assessed by having one observer remeasure after 4–8 weeks. Interobserver variability was evaluated by a second observer who was blinded to the first observer’s measurements.

### Outcomes

Patients were followed until 15 March 2022. The follow-up on outcomes started the day after the comprehensive baseline echocardiography measurements. Follow-up data were collected through hospital visits or telephone contacts by the investigator who was blinded to clinical details and echocardiography data. The primary end point was a combined outcome of heart failure-related hospitalization or death.

### Statistical analysis

All continuous variables are presented as means with 95% confidence intervals (CIs), and categorical variables are presented as numbers and percentages. Comparisons of clinical and echocardiographic characteristics between HFpEF patients with and without T2DM were performed using chi-square tests for categorical data and two-sample *t*-test or a Mann-Whitney U test for continuous variables. The prognostic value of LA function parameters was assessed using univariate and multivariate Cox regression to obtain hazard ratio (HR) and 95% CI. Survival curves were obtained using the Kaplan-Meier analysis and compared using the log-rank test. All statistical analyses were performed using SPSS version 24 (IBM SPSS Statistics, Chicago, IL). *P*-value < 0.05 was considered to indicate statistical significance.

## Results

### Baseline characteristics

The baseline clinical and echocardiographic characteristics of the 164 HFpEF patients are summarized in Table 1. T2DM was identified in 61 patients (37%), and the remaining 103 patients (63%) were classified as non-T2DM patients. There were no significant differences in age, gender, BMI, systolic blood pressure, diastolic blood pressure, heart rate, medications use, and laboratory findings between the two groups, except for more SGLT-2i use, increased BNP or NT-proBNP, and reduced high-density lipoprotein levels in HFpEF patients with T2DM. HFpEF patients with T2DM suffered more frequently from NYHA functional class III or IV symptoms (41% vs. 17%, *P* = 0.001). However, frequencies of comorbidities including hypertension, hypercholesterolemia, and coronary artery disease were similarly distributed between the two groups (all *P* > 0.05). In addition, LV global longitudinal strain in HFpEF patients with T2DM was significantly impaired (-18.6 [17.8–19.4]% vs. -19.7 [19.2–20.2]%, *P* = 0.018), and E/e’ was higher (14.7 [13.7–15.7] vs. 12.5 [11.7–13.2], *P* = 0.001) than of those without T2DM. In contrast, E/A, LVEF, LV, and LA volumes for the two groups were similar (all *P* > 0.05).

**TABLE 1:** Demographic and echocardiographic parameters in HFpEF patients according to diabetes status.

	All patients (*n* = 164)	HFpEF patients with T2DM (*n* = 61)	HFpEF patients without T2DM (*n* = 103)	*P*-value
**Demographics**				
Age (years)	63 (61–64)	61 (59.0–63.6)	63 (61–65)	0.211
Sex (female)	77 (47)	28 (46)	49 (48)	0.840
BMI (kg/m^2^)				0.280
Underweight (<18.5 kg/m^2^)	6 (4)	3 (5)	3 (3)	
Normal (18.5–24.9 kg/m^2^)	89 (54)	32 (53)	57 (55)	
Overweight (25–29.9 kg/m^2^)	65 (40)	22 (36)	43 (42)	
Obese (30 kg/m^2^)	4 (2)	4 (6)	0 (0)	
Systolic blood pressure (mmHg)	135 (132–138)	137 (133–142)	134 (130–137)	0.204
Diastolic blood pressure (mmHg)	78 (76–80)	79 (75–82)	78 (75–80)	0.550
Heart rate (bpm)	75 (73–76)	76 (74–79)	73 (71–76)	0.109
NYHA functional class ≥ III, n (%)	43 (26)	25 (41)	18 (17)	0.001
Hypertension, n (%)	128 (78)	50 (82)	78 (76)	0.350
Hypercholesterolemia, n (%)	41 (25)	16 (26)	25 (24)	0.780
Coronary artery disease, n (%)	103 (63)	43 (71)	60 (58)	0.120
**Medication use**				
Beta-blockers, n (%)	110 (67)	40 (66)	70 (68)	0.750
Aspirin, n (%)	93 (57)	36 (59)	57 (55)	0.650
ACEI/ARB, n (%)	104 (63)	42 (69)	62 (60)	0.230
ARNI, n (%)	35 (21)	14 (23)	21 (20)	0.699
Clopidogrel, n (%)	58 (35)	22 (36)	36 (35)	0.890
Diuretics, n (%)	52 (32)	23 (38)	29 (28)	0.200
Statin, n (%)	133 (81)	50 (82)	83 (81)	0.830
CCB, n (%)	103 (63)	40 (66)	63 (61)	0.570
Aldosterone antagonists, n (%)	17 (10)	4 (7)	13 (13)	0.293
SGLT-2i	31 (19)	30 (49)	1 (1)	<0.001
**Laboratory findings**				
eGFR (mL/min/1.73 m^2^)	70.3 (65.2–75.3)	63.9 (54.7–73.0)	74.1 (68.1–80.0)	0.550
Total cholesterol (mmol/L)	4.1 (3.9–4.3)	4.0 (3.6–4.3)	4.2 (3.9–4.5)	0.340
Triglyceride (mmol/L)	1.4 (1.2–1.6)	1.6 (1.2–2.0)	1.3 (1.1–1.5)	0.150
High-density lipoprotein (mmol/L)	1.2 (1.1 –1.2)	1.0 (0.9–1.1)	1.2 (1.2–1.3)	<0.001
Low-density lipoprotein (mmol/L)	2.4 (2.2–2.6)	2.3 (2.0–2.6)	2.4 (2.2–2.7)	0.490
BNP (pg/mL)	187.9 (150.9–224.8) (*n* = 108)	263.4 (186.2–340.7) (*n* = 38)	143.1 (107.7–178.5) (*n* = 70)	<0.001
NT-proBNP (pg/mL)	2051.4 (272.1–3830.7) (*n* = 56)	4126.4 (123.3–8129.4) (*n* = 23)	605.2 (295.5–915.0) (*n* = 33)	0.039
**Echocardiographic measures**				
LVEF (%)	59.7 (58.8–60.6)	58.6 (57.0–60.3)	60.3 (59.2–61.3)	0.100
LV Global longitudinal strain (%)	-19.3 (18.8–19.7)	-18.6 (17.8–19.4)	-19.7 (19.2–20.2)	0.018
LV end-diastolic volume (mL)	106.7 (100.3–113.2)	111.3 (99.3–123.4)	104.0 (96.5–111.5)	0.280
LV end-diastolic volume index (mL/m^2^)	62.6 (59.2–66.0)	64.4 (58.4–70.3)	61.5 (57.2–65.6)	0.420
LV end-systolic volume (mL)	44.5 (40.9–48.2)	48.1 (41.1–55.1)	42.4 (38.3–46.5)	0.160
LV end-systolic volume index (mL/m^2^)	26.1 (24.1–28.0)	27.7 (24.1–31.3)	25.1 (22.7–27.4)	0.210
E (cm/s)	0.86 (0.82–0.89)	0.87 (0.81–0.94)	0.84 (0.79–0.90)	0.490
A (cm/s)	0.92 (0.88–0.96)	0.93 (0.86–0.99)	0.91 (0.86–0.97)	0.790
E/A	1.06 (0.95–1.17)	1.03 (0.90–1.15)	1.08 (0.93–1.24)	0.620
E/e’ average ratio	13.3 (12.7–14.0)	14.7 (13.7–15.7)	12.5 (11.7–13.2)	0.001
LA volume (mL)	87.7 (83.7–91.7)	89.9 (82.0–97.8)	86.4 (82.0–90.8)	0.440
LA volume index (mL/m^2^)	51.8 (49.6–53.9)	52.5 (48.3–56.6)	51.3 (48.9–53.7)	0.640

Values are shown as means (95% CIs) or numbers (percentage). ACEI, angiotensin-converting enzyme inhibitor; ARB, angiotensin II receptor blocker; ARNI, angiotensin receptor enkephalinase inhibitor; BMI, body mass index; CCB, calcium channel blocker; EF, ejection fraction; eGFR, estimated glomerular filtration rate; HFpEF, heart failure with preserved ejection fraction; LV, left ventricular; LA, left atrial; SGLT-2i, sodium-glucose cotransporter-2 inhibitor; T2DM, type 2 diabetes mellitus; NYHA, New York Heat Association.

### Comparisons of LA function and stiffness in HFpEF patients with and without T2DM

**FIGURE 1:**
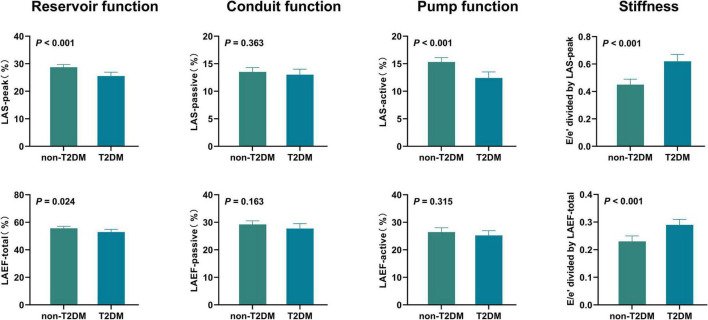
Unadjusted comparisons of LA function and stiffness between HFpEF patients with and without T2DM HFpEF, heart failure with preserved ejection fraction; LA, left atrial; LAS, LA strain; LAEF, LA ejection fraction; T2DM, type 2 diabetes mellitus.

Unadjusted comparisons of LA function and stiffness between HFpEF patients with and without T2DM are shown in Table 2 and Figure 1. LAS-peak and LAEF-total (reflecting LA reservoir function) and LAS-active (reflecting LA pump function) were significantly lower in HFpEF patients with T2DM than those without T2DM (all *P* < 0.05). Moreover, E/e’ divided by LAS-peak and E/e’ divided by LAEF-total (reflecting LA stiffness) in HFpEF patients with T2DM were higher than those without T2DM (both *P* < 0.001). However, LAS-passive and LAEF-passive (measurement of LA conduit function) and LAEF-active (another measurement of LA pump function) had no significant differences between HFpEF patients with and without T2DM.

**TABLE 2:** Unadjusted comparisons of LA function and stiffness between HFpEF patients with and without T2DM.

Variables	HFpEF patients with T2DM (*n* = 61)	HFpEF patients without T2DM (*n* = 103)	*P*-value
**Reservoir function**			
LAS-peak (%)	25.5 (24.1–26.9)	28.7 (27.7–29.8)	<0.001
LAEF-total (%)	52.9 (50.9–54.8)	55.6 (54.2–57.0)	0.024
**Conduit function**			
LAS-passive (%)	13.0 (12.0–14.0)	13.5 (12.8–14.3)	0.363
LAEF-passive (%)	27.7 (25.9–29.4)	29.2 (27.9–30.4)	0.163
**Pump function**			
LAS-active (%)	12.4 (11.3–13.5)	15.3 (14.5–16.1)	<0.001
LAEF-active (%)	25.2 (23.4–26.9)	26.4 (24.9–28.0)	0.315
**Stiffness**			
E/e’ divided by LAS-peak	0.619 (0.566–0.672)	0.451 (0.410–0.492)	<0.001
E/e’ divided by LAEF-total	0.288 (0.265–0.311)	0.229 (0.211–0.247)	<0.001

Numbers are shown as means (95% CIs). HFpEF, heart failure with preserved ejection fraction; LA, left atrial; LAS, left atrial strain; LAEF, left atrial ejection fraction; T2DM, type 2 diabetes mellitus.

**FIGURE 2:**
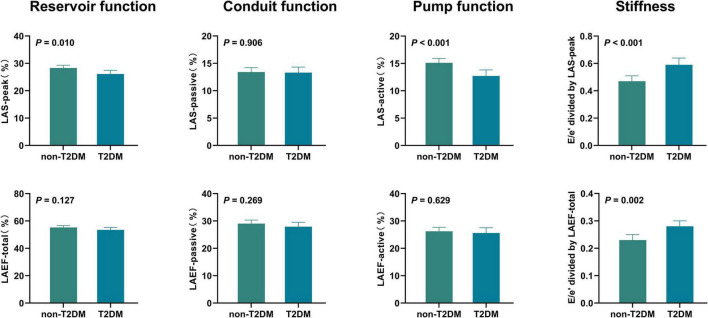
Adjusted comparisons of LA function and stiffness between HFpEF patients with and without T2DM HFpEF, heart failure with preserved ejection fraction; LA, left atrial; LAS, LA strain; LAEF, LA ejection fraction; T2DM, type 2 diabetes mellitus. Adjusted for age, sex, body mass index, heart rate, systolic blood pressure, LV global longitudinal strain, and (or) E/e’.

After adjusting for age, sex, BMI, heart rate, systolic blood pressure, LV global longitudinal strain, and (or) E/e’, differences persisted in LA reservoir function (measured by LAS-peak), LA pump function (measured by LAS-active), and LA stiffness between HFpEF patients with and without T2DM (all *P* < 0.05) (Table 3 and Figure 2).

**TABLE 3:** Adjusted comparisons of LA function and stiffness between HFpEF patients with and without T2DM.

Variables	HFpEF patients with T2DM (*n* = 61)	HFpEF patients without T2DM (*n* = 103)	*P*-value
**Reservoir function**			
LAS-peak (%)	26.1 (24.8–27.4)	28.3 (27.4–29.3)	0.010
LAEF-total (%)	53.5 (51.6–55.3)	55.2 (53.9–56.6)	0.127
**Conduit function**			
LAS-passive (%)	13.3 (12.3–14.3)	13.4 (12.6–14.1)	0.906
LAEF-passive (%)	27.9 (26.2–29.5)	29.0 (27.8–30.3)	0.269
**Pump function**			
LAS-active (%)	12.7 (11.7–13.8)	15.1 (14.3–15.9)	<0.001
LAEF-active (%)	25.6 (23.6–27.5)	26.2 (24.7–27.7)	0.629
**Stiffness**			
E/e’ divided by LAS-peak	0.594 (0.545–0.644)	0.466 (0.428–0.503)	<0.001
E/e’ divided by LAEF-total	0.280 (0.257–0.303)	0.234 (0.216–0.251)	0.002

Numbers are shown as means (95% CIs). HFpEF, heart failure with preserved ejection fraction; LA, left atrial; LAS, left atrial strain; LAEF, left atrial ejection fraction; T2DM, type 2 diabetes mellitus. Adjusted for age, sex, body mass index, heart rate, systolic blood pressure, LV global longitudinal strain, and (or) E/e’.

### Prognostic value of LA function

**FIGURE 3:**
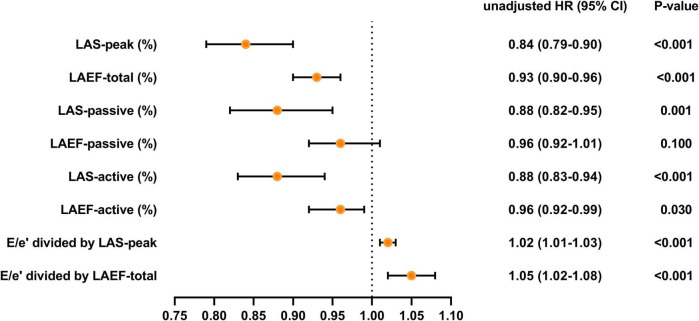
Unadjusted HR for various measures of LA function and stiffness HR, hazard ratio; LA, left atrial; LAS, LA strain; LAEF, LA ejection fraction.

**FIGURE 4:**
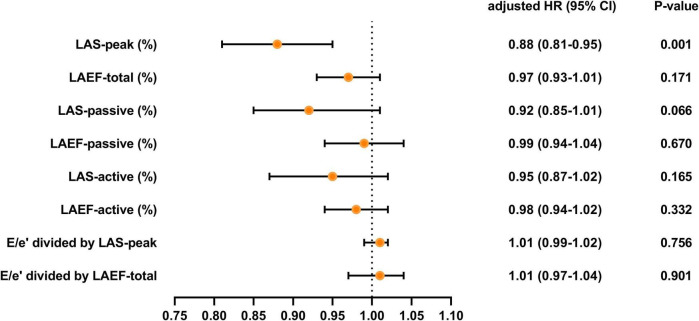
Adjusted HR for various measures of LA function and stiffness HR, hazard ratio; LA, left atrial; LAS, LA strain; LAEF, LA ejection fraction. Models were adjusted for BMI, diabetes, LV global longitudinal strain, LA volume index, and (or) E/e’.

Over a median duration of 13.7 (IQR 10.7–14.1) months, 46 patients (28.1%) experienced the primary composite end point, including 40 (24.4%) hospitalized for heart failure and 6 (3.7%) died during follow-up. Table 4, Figures 3 and 4 demonstrate the results of proportional hazards (Cox) models in which LA function and stiffness were assessed as predictors of adverse events.

**TABLE 4:** Univariate and multivariate Cox proportional hazards analysis.

	Unadjusted		Adjusted*	
		
	HR (95% CI)	*P* value	HR (95% CI)	*P*-value
**Reservoir function**				
LAS-peak (%)	0.84 (0.79–0.90)	<0.001	0.88 (0.81–0.95)	0.001
LAEF-total (%)	0.93 (0.90–0.96)	<0.001	0.97 (0.93–1.01)	0.171
**Conduit function**				
LAS-passive (%)	0.88 (0.82–0.95)	0.001	0.92 (0.85–1.01)	0.066
LAEF-passive (%)	0.96 (0.92–1.01)	0.100	0.99 (0.94–1.04)	0.670
**Pump function**				
LAS-active (%)	0.88 (0.83–0.94)	<0.001	0.95 (0.87–1.02)	0.165
LAEF-active (%)	0.96 (0.92–0.99)	0.030	0.98 (0.94–1.02)	0.332
**Stiffness**				
E/e’ divided by LAS-peak	1.02 (1.01–1.03)	<0.001	1.01 (0.99–1.02)	0.756
E/e’ divided by LAEF-total	1.05 (1.02–1.08)	<0.001	1.01 (0.97–1.04)	0.901

Numbers are shown as means (95% CI). HR, hazard ratio; LAS, left atrial strain; LAEF, left atrial ejection fraction. *Adjusted for BMI, diabetes, LV global longitudinal strain, LA volume index, and (or) E/e’.

In unadjusted analyses (Table 4 and Figure 3), LAS-peak (HR: 0.84; 95% CI: 0.79–0.90; *P* < 0.001), LAEF-total (HR: 0.93; 95% CI: 0.90–0.96; *P* < 0.001), LAS-active (HR: 0.88; 95% CI: 0.83–0.94; *P* < 0.001), LAEF-active (HR: 0.96; 95% CI: 0.92–0.99; *P* = 0.030), E/e’ divided by LAS-peak (HR: 1.02; 95% CI: 1.01–1.03; *P* < 0.001), and E/e’ divided by LAEF-total (HR: 1.05; 95% CI: 1.02–1.08; *P* < 0.001) were significantly predictive of adverse events. Although the LAS-passive (HR: 0.88; 95% CI: 0.82–0.95; *P* = 0.001) was significantly predictive of adverse events, LAEF-passive was not a predictor of clinical events. The results of univariate analysis evaluating baseline clinical and other echocardiographic variables associated with combined endpoint events are reported in Supplementary Table 1.

In analyses that adjusted for BMI, diabetes, LV global longitudinal strain, LA volume index, and (or) E/e’ (Table 4 and Figure 4), LAEF-total, LAS-passive, LAS-active, LAEF-active, and LA stiffness were not significant predictors of adverse events. LAS-peak (HR: 0.88; 95% CI: 0.81–0.95; *P* = 0.001) remained significantly predictive of the risk of adverse events.

### Association of T2DM and LA function with outcome for HFpEF patients

**FIGURE 5:**
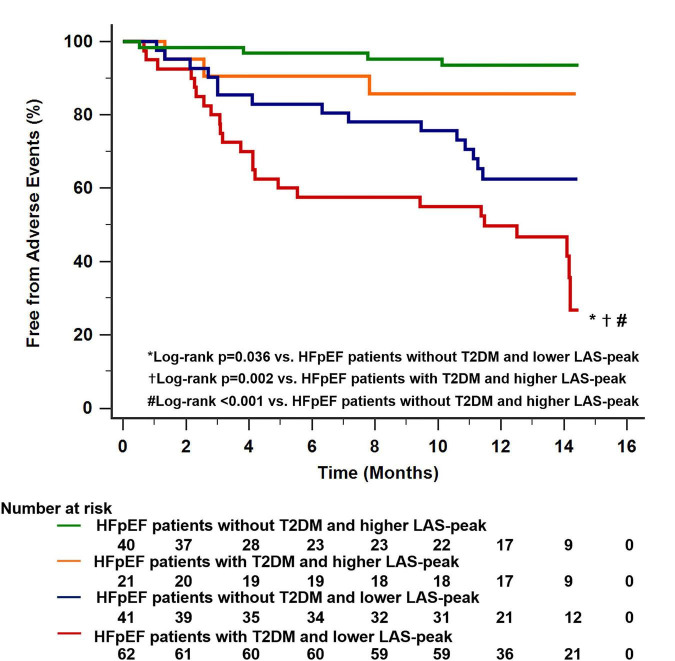
Dividing all HFpEF patients into two main groups using the median value of LAS-peak (27.2%) identified 40 patients with HFpEF with T2DM and low LAS-peak. This characteristic was associated with worse long-term outcomes compared with the other subgroups. HFpEF, heart failure with preserved ejection fraction; LAS, left atrial strain; T2DM, type 2 diabetes mellitus.

All HFpEF patients were classified into two groups using the median value of LAS-peak (27.2%). There were 40 HFpEF patients with T2DM and low LAS-peak (<27.2%). This feature was associated with a worse clinical outcome than for the other subgroups (log-rank *P* = 0.037 vs. HFpEF patients without T2DM and low LAS-peak; log-rank *P* = 0.002 vs. HFpEF patients with T2DM and high LAS-peak; log-rank *P* < 0.001 vs. HFpEF patients without T2DM and high LAS-peak; Figure 5).

### Intra- and inter-observer variability

Intra-observer and inter-observer reproducibility of LA strain by 2D-STE is shown in Supplementary Table 2. LAS-peak, LAS-active, and LAS-passive exhibited good reproducibility, as reflected by high ICC. Bland-Altman analysis demonstrated high intra-observer and inter-observer agreement, with small bias and narrow limits of agreement.

## Discussion

In this prospective study, we comprehensively compared LA function and stiffness between HFpEF subjects with and without T2DM. We demonstrated that LA reservoir function (measured by LAS-peak) and LA pump function (measured by LAS-active) were significantly impaired in the HFpEF patients with T2DM compared with those without T2DM, even after adjustment for potential confounders. In addition, LA stiffness was higher in HFpEF patients with T2DM than of those without T2DM. More important, LAS-peak was a powerful predictor of heart failure hospitalization or death, independently of other clinical and echocardiographic parameters. In contrast, other LA function and stiffness parameters did not independently predict the risk of adverse events.

### LA function and stiffness in HFpEF with and without T2DM

Although the LA dysfunction in HFpEF has been demonstrated in previous studies (15–21, 24), the role of all three phases of LA function in HFpEF patients with T2DM is less well-established. To the best of our knowledge, there is only one report on LA function in HFpEF patients with and without T2DM from a single center and small sample study (12). However, in this publication from Ljubica et al., LA function was incompletely assessed (only LA reservoir and pump function were evaluated by strain), and potential confounders were not adjusted. Moreover, they did not investigate the prognostic value of the LA function and stiffness in HFpEF patients with or without diabetes mellitus. In this study, we comprehensively assessed LA function using both volumetric measures (LAEF-total, LAEF-active, and LAEF-passive) and strain-based measures (LAS-peak, LAS-passive, and LAS-active) based on 2D-STE. After adjusting for potential confounders, we found that LAS-peak and LAS-active remained reduced in HFpEF patients with T2DM compared with subjects without T2DM. There were several hypotheses that showed mechanisms of T2DM on LA structure and function (36, 37). First, sustained hyperglycemia induces interstitial fibrosis not only in the LV but also in LA. Second, hyperglycemia is related to enhanced pro-fibrotic signaling molecules that provoke collagen synthesis by cardiac fibroblasts implying that these factors can promote atrial fibrosis in DM. In addition, given that several recent studies suggested that worse LV longitudinal systolic function (38, 39), elevated LV filling pressures (40, 41), and LA size (42) may contribute to LA dysfunction. To avoid the influence of these confounding factors, we adjusted them. In addition, LA stiffness, which is related to LA reservoir function and LV filling pressure, increases with LA remodeling and is recognized as a further indicator of LA performance (27). Nonetheless, LA stiffness in HFpEF patients with and without T2DM has not been described. In our study, we also compared the LA stiffness between the two groups. We found that the LA stiffness was consistently increased in HFpEF subjects with T2DM, both before and after adjustment. Therefore, we thought that T2DM may negatively affect the LA function and stiffness.

### Prognostic value of left atrium function in heart failure with preserved ejection fraction with and without type 2 diabetes mellitus

The LA reservoir function is a predictor of poor outcomes in various cardiovascular diseases, particularly in heart failure with reduced ejection fraction and stable coronary heart disease (43–46). However, there is less evidence on the prognostic relevance of LA function in HFpEF. Moreover, data on the prognostic value regarding LA function in HFpEF are controversial. Freed et al. assessed LA function measured by strain in HFpEF patients who were followed for a median of 13.8 months, and 115 experienced composite events of hospitalization or death. They revealed that LA reservoir strain and conduit strain remained prognostic after adjustment for atrial fibrillation, LA volume, LV mass, and the MAGGIC risk score (25). In contrast, Santos et al. evaluated LA function measured by volume and strain in symptomatic HFpEF patients who were followed for a median of 31 months (91 composite events). They showed that LA reservoir function was not an independent predictor of mortality in HFpEF (26). In our study, we found that decreased LAS-peak was significantly associated with the risk of composite all-cause mortality or heart failure-related hospitalization, even after adjusting for demographic and clinical characteristics, LV global longitudinal strain, and E/e’. This result is partially consistent with the findings of Santos et al. In addition, T2DM is an independent risk factor for cardiovascular disease and its associated mortality (6–8), and interest in the assessment of risk stratification for HFpEF patients with T2DM has remained strong. In this study, Kaplan-Meier analysis showed that HFpEF patients with T2DM and low LAS-peak (<27.2%) had a significantly increased risk of poor outcomes (log-rank *P* < 0.001). Therefore, LAS-peak may be essential for risk stratification in HFpEF patients with and without T2DM.

The LA stiffness has been recognized as a novel method that estimates LA diastolic function. Recently, Khurram et al. enrolled 219 patients with AF referred for ablation. After a median follow-up of 10 months, 40 patients had recurrence after AF ablation. Patients with recurrence had higher LA stiffness than those without recurrence. Therefore, Khurram et al. reported that LA stiffness is a strong independent predictor of recurrence after ablation (28). Furthermore, Ibadete et al. revealed that LA stiffness is associated with poor clinical outcomes in patients with heart failure and reduced ejection fraction (29, 30). In our study, LA stiffness was a significant predictor of clinical events in univariate analysis, However, LA stiffness was not a significant predictor of adverse events after adjusting BMI, diabetes, LV global longitudinal strain, and LA volume index. The predictive value of LA stiffness still needed multicenter and large sample data to confirm.

### Limitation

Certain limitations should also be considered. First, this study covered a relatively limited number of patients in a single-center study. Our findings require to be tested in future multicenter studies with larger patient populations. Furthermore, the relatively small number of patients and clinical events limited the power of our ability to adjust for confounders in the Cox model for the combined endpoint. Second, we did not have access to data on the severity or duration of diabetes. Third, subjects with atrial fibrillation and poor echocardiographic image quality were excluded from the analysis. Therefore, there was a risk of selection bias. However, we were able to perform speckle-tracking analysis on the majority of the study participants, and reproducibility was excellent.

## Conclusion

Our study comprehensively evaluates the LA function and stiffness and investigates their prognostic significance in HFpEF patients with and without T2DM. We demonstrate that strain-based LA reservoir and pump function are impaired, whereas LA stiffness is increased in HFpEF patients with T2DM compared with those without T2DM. More important, LAS-peak is a powerful predictor of heart failure hospitalization or death, independently of other clinical and echocardiographic parameters. Therefore, a comprehensive assessment of LA function using 2D STE may be essential for risk stratification in HFpEF patients with and without T2DM.

## Data availability statement

The raw data supporting the conclusions of this article will be made available by the authors, without undue reservation.

## Ethics statement

This study was approved by the Ethics Committee of Wuhan Union Hospital, Tongji Medical College, Huazhong University of Science and Technology, and was performed in accordance with the Declaration of Helsinki (Ethics No. 0650-01). The patients/participants provided their written informed consent to participate in this study. Written informed consent was obtained from the individual(s) for the publication of any potentially identifiable images or data included in this article.

## Author contributions

SZ, YiL, YZ, MX, YuL, and LZ conceived the idea and designed this study and drafted the manuscript. SZ, YiL, YZ, MQ, and LG were responsible for the analysis and interpretation of data. SZ, YiL, YZ, GW, MQ, LG, MJ, MX, YuL, and LZ revised the manuscript critically for important intellectual content. SZ, YiL, YZ, GW, MX, YuL, and LZ gave the final approval of the manuscript submitted. All authors contributed to the article and approved the submitted version.
