# Supplementary material for: Prognostic relevance of left atrial function and stiffness in heart failure with preserved ejection fraction patients with and without diabetes mellitus
Source: Front Cardiovasc Med. 2022 Sep 14;9:947639. doi: 10.3389/fcvm.2022.947639 (PMC9515445; doi:10.3389/fcvm.2022.947639)
Supplement: Supplementary file 1 [file Data_Sheet_1.docx]

| **Supplemental Table 1. Univariate cox proportional-hazards analysis** **evaluating baseline clinical and other echocardiographic variables associations with combined endpoint events** | | |
| --- | --- | --- |
|  | **Unadjusted** | |
| **Variables** | **HR (95% CI)** | **p Value** |
| **Demographics** |  |  |
| Age (years) | 0.98 (0.95-1.01) | 0.112 |
| Sex (female) | 0.74 (0.41-1.34) | 0.321 |
| BMI, kg/m2 | 1.09 (1.01-1.19) | 0.034 |
| Systolic blood pressure (mmHg) | 1.01 (0.99-1.02) | 0.545 |
| Diastolic blood pressure (mmHg) | 1.02 (1.00-1.05) | 0.075 |
| Heart rate (bpm) | 1.00 (0.97-1.03) | 0.878 |
| T2DM | 0.37 (0.20-0.66) | 0.001 |
| Hypertension, n (%) | 0.85 (0.41-1.77) | 0.671 |
| Hypercholesterolemia (n, %) | 0.84 (0.44-1.60) | 0.593 |
| Coronary artery disease, n (%) | 0.76 (0.41-1.41) | 0.385 |
| **Medication use** |  |  |
| Beta-blockers, n (%) | 0.77 (0.40-1.46) | 0.417 |
| Aspirin | 0.74 (0.41-1.35) | 0.321 |
| ACEI/ARB | 0.72 (0.38-1.34) | 0.298 |
| ARNI | 0.32 (0.18-0.59) | <0.001 |
| Clopidogrel | 0.75 (0.42-1.35) | 0.338 |
| Diuretics | 0.51 (0.28-0.90) | 0.021 |
| Statin | 1.07 (0.51-2.21) | 0.862 |
| CCB | 0.55 (0.28-1.05) | 0.071 |
| Aldosterone antagonists | 0.71 (0.30-1.68) | 0.440 |
| SGLT2I | 0.73 (0.37-1.44) | 0.366 |
| **Laboratory findings** |  |  |
| eGFR (mL/min/1.73 m^2^) | 0.99 (0.98-1.00) | 0.005 |
| Total cholesterol, mmol/L | 0.80 (0.61-1.06) | 0.115 |
| Triglyceride, mmol/L | 1.25 (1.01-1.55) | 0.044 |
| High-density lipoprotein, mmol/L | 0.18 (0.07-0.51) | 0.001 |
| Low-density lipoprotein, mmol/L | 0.68 (0.48-0.97) | 0.033 |
| BNP, pg/ml (n=108) | 1.001 (1.000-1.002) | 0.005 |
| NT-proBNP, pg/ml (n=56) | 1.000 (1.000-1.001) | 0.001 |
| **Echocardiographic measures** |  |  |
| LVEF (%) | 0.94 (0.90-0.99) | 0.008 |
| LV GLS (%) | 1.18 (1.08-1.29) | <0.001 |
| LV EDV (mL) | 1.01 (1.01-1.02) | 0.001 |
| LVEDV index (ml/m^2^) | 1.02 (1.01-1.03) | 0.002 |
| LVESV (mL) | 1.02 (1.01-1.03) | <0.001 |
| LVESV index (ml/m^2^) | 1.03 (1.10-1.05) | 0.001 |
| E (cm/s) | 0.81 (0.26-2.46) | 0.703 |
| A (cm/s) | 1.39 (0.46-4.16) | 0.56 |
| E/A | 0.93 (0.58-1.50) | 0.771 |
| E/e’ average ratio | 1.05 (1.03-1.07) | 0.048 |
| LAV (ml) | 1.02 (1.01-1.03) | <0.001 |
| LAV index (ml/m^2^) | 1.03 (1.01-1.06) | 0.001 |

Values are shown as means (95% CIs) or number (percentage). ACEI, angiotensin-converting enzyme inhibitor; ARB, angiotensin II receptor blocker; Angiotensin receptor enkephalinase inhibitor (ARNI); BMI, body mass index; CCB, calcium channel blocker; EF, ejection fraction; eGFR, estimated glomerular filtration rate; HFpEF, heart failure with preserved ejection fraction; LV, left ventricular; LA, left atrial; SGLT-2i, sodium-glucose cotransporter-2 inhibitor; T2DM, type 2 diabetes mellitus; NYHA, New York Heat Association.

| **Supplemental Table 2. Intra-observer and Inter-observer Reproducibility** | | | |
| --- | --- | --- | --- |
|  | **ICC (95%CI)** | **Bias** | **Limits of agreement** |
| **Intra-observer** |  |  |  |
| LAS-peak (%) | 0.97 (0.93-0.99) | -0.28 | -2.15 to1.59 |
| LAS-passive (%) | 0.78 (0.52-0.91) | -0.39 | -3.40 to 2.62 |
| LAS-active (%) | 0.85 (0.65-0.93) | -0.06 | -3.35 to 3.23 |
| **Inter-observer** |  |  |  |
| LAS-peak (%) | 0.95 (0.88-0.98) | -1.13 | -3.67 to 1.30 |
| LAS-passive (%) | 0.79 (0.54-0.91) | -0.60 | -3.81 to 2.61 |
| LAS-active (%) | 0.79 (0.54-0.91) | -0.76 | -4.54 to 3.03 |

Numbers are shown as means (95% CIs); ICC = intraclass correlation coefficient; LA, left atrial; LAS, LA strain.
